# Maternal dietary intake of nitrates, nitrites and nitrosamines and selected birth defects in offspring: a case-control study

**DOI:** 10.1186/1475-2891-12-34

**Published:** 2013-03-21

**Authors:** John C Huber, Jean D Brender, Qi Zheng, Joseph R Sharkey, Ann M Vuong, Mayura U Shinde, John S Griesenbeck, Lucina Suarez, Peter H Langlois, Mark A Canfield, Paul A Romitti, Peter J Weyer

**Affiliations:** 1 Department of Epidemiology and Biostatistics, The Texas A&M Health Science Center School of Rural Public Health, MS 1266 TAMU, College Station, TX 77843-1266, USA; 2 Department of Health Promotion & Community Health Sciences, The Texas A&M Health Science Center School of Rural Public Health, MS 1266 TAMU, College Station, TX 77843-1266, USA; 3 111 Marine Expeditionary Force, Okinawa, Japan; 4 Texas Department of State Health Services, PO Box 149347, Austin, Texas 78714-9347, USA; 5 Department of Epidemiology, The University of Iowa College of Public Health, C21-E GH, 200 Hawkins Drive, Iowa City, Iowa 52242, USA; 6 The University of Iowa Center for Health Effects of Environmental Contamination, N202 Oakdale Hall, Iowa City, Iowa 52242, USA

**Keywords:** Congenital malformation, Diet, Nitrate, Nitrite, Nitrosamine, Pregnancy

## Abstract

**Background:**

Dietary intake of nitrates, nitrites, and nitrosamines can increase the endogenous formation of N-nitroso compounds in the stomach. Results from animal studies suggest that these compounds might be teratogenic. We examined the relationship between maternal dietary intake of nitrates, nitrites (including plant and animal sources as separate groups), and nitrosamines and several types of birth defects in offspring.

**Methods:**

For this population-based case–control study, data from a 58-question food frequency questionnaire, adapted from the short Willett Food Frequency Questionnaire and administered as part of the National Birth Defects Prevention Study (NBDPS), were used to estimate daily intake of dietary nitrates, nitrites, and nitrosamines in a sample of 6544 mothers of infants with neural tube defects (NTD)s, oral clefts (OC)s, or limb deficiencies (LD)s and 6807 mothers of unaffected control infants. Total daily intake of these compounds was divided into quartiles based on the control mother distributions. Odds ratios (OR)s and 95% confidence intervals (CI)s were estimated using logistic regression; estimates were adjusted for maternal daily caloric intake, maternal race-ethnicity, education, dietary folate intake, high fat diet (> 30% of calories from fat), and state of residence.

**Results:**

While some unadjusted ORs for NTDS had 95% (CI)s that excluded the null value, none remained significant after adjustment for covariates, and the effect sizes were small (adjusted odds ratios [aOR] <1.12). Similar results were found for OCs and LDs with the exception of animal nitrites and cleft lip with/without cleft palate (aORs and CIs for quartile 4 compared to quartile 1 =1.24; CI=1.05-1.48), animal nitrites and cleft lip (4th quartile aOR=1.32; CI=1.01-1.72), and total nitrite and intercalary LD (4th quartile aOR=4.70; CI=1.23-17.93).

**Conclusions:**

Overall, odds of NTDs, OCs or LDs did not appear to be significantly associated with estimated dietary intake of nitrate, nitrite, and nitrosamines.

## Background

Findings from a few epidemiologic studies have suggested that prenatal exposure to nitrates (from drinking water) [1] and nitrites, especially in conjunction with nitrosatable drugs [2,3], are associated with neural tube defects (NTD)s. About five percent of nitrate is converted to nitrite after ingestion [4]. This nitrite along with dietary nitrite can react with amines and amides in an acidic environment such as that found in the stomach to form nitrosamines and nitrosamides [5]. Results from several animal studies have indicated that various *N*-nitroso compounds may be teratogenic [6-11]. In hamsters, nitrosamines have been noted to cross the placental barrier [12], even at low doses [13].

In our previous studies of maternal nitrosatable drug exposure among study participants in the National Birth Defects Prevention Study (NBDPS), we found that 24% of the control women (mothers who gave birth to babies without major congenital malformations) took one or more nitrosatable drugs during the first trimester [14]. These drugs have secondary amines, tertiary amines, or amides as part of their molecular structures. In the NBDPS study population, maternal exposures to nitrosatable drugs were associated with neural tube defects [3], limb deficiencies [15], and some types of heart defects [15]. We noted the strongest associations between maternal exposure to these drugs and neural tube defects, conotruncal heart defects, atrioventricular septal defects, single ventricle, and cleft palate in offspring of women with the highest estimated total nitrite intake.

Studies investigating the relation between maternal dietary exposures to nitrates, nitrites and nitrosamines and birth defects have involved relatively small sample sizes and were restricted to NTDs [1,2]. Furthermore, these studies grouped plant and animal sources of nitrite together. However, some plant sources of nitrites, such as cereals, are fortified with vitamins including vitamin C, while animal sources of nitrites are less likely to contribute significantly to vitamin C intake. Vitamin C is a well-documented inhibitor of nitrosation and the formation of N-nitroso compounds in the stomach [16]. This study examines the relationship between maternal exposure to dietary nitrates, nitrites (including plant and animal sources as separate groups), and nitrosamines and several types of birth defects including NTDs, orofacial clefts, and limb malformations in a large, population-based case–control study.

## Methods

### Study design and sample

We used data from the National Birth Defects Prevention Study (NBDPS) [17] to address the study objectives. Funded by the Centers for Disease Control and Prevention, the NBDPS is a population-based, case–control study that includes sites in Georgia, Arkansas, California, Iowa, Massachusetts, New Jersey, New York, North Carolina, Texas and Utah. Mothers who gave birth to babies without congenital malformations (controls, n=6807) were compared with case mothers (n=6544) whose pregnancies were affected by orofacial clefts, limb deficiencies or neural tube defects. Eligible participants had estimated delivery dates from October 1, 1997 through December 31, 2005. Participants were excluded if their self-reported daily caloric intake was below 500 calories or greater than 5000 calories. The demographic characteristics of the mothers are described in Table 1.

**Table 1:** Selected maternal characteristics of birth defect cases and controls in the National Birth Defects Prevention Study, 1997-2005

**Characteristics of participants**		**Control**		**Neural tube defects**		**Limb deficiencies**		**Oral cleft defects**	
		**N = 6698**		**N = 1170**		**N = 693**		**N = 2727**	
		**N**	**%**	**N**	**%**	**N**	**%**	**N**	**%**
Race-ethnicity^a,b,c^									
	Non-Hispanic White	3,989	59.6	596	50.9	388	56.0	1,708	62.6
	Non-Hispanic Black	758	11.3	108	9.2	71	10.3	173	6.3
	Hispanic	1,464	21.9	377	32.2	190	27.4	640	23.5
	Asian/Pacific Islander	197	2.9	25	2.1	14	2.0	81	3.0
	Native American/Alaskan	29	0.4	7	0.6	3	0.4	23	0.8
	Other	233	3.5	53	4.5	27	3.9	94	3.5
	Missing	28	0.4	4	0.3	0	0.0	8	0.3
Maternal Education (years) ^a,c^									
	<9 years	325	4.9	95	8.1	33	4.8	158	5.8
	9-11 years	770	11.5	149	12.7	81	11.7	350	12.8
	12 years	1,627	24.3	332	28.4	185	26.7	761	27.9
	13-15 years	1,784	26.6	328	28.0	197	28.4	724	26.6
	16 years or more	2,096	31.3	252	21.5	188	27.1	708	26.0
	Missing	96	1.4	14	1.2	9	1.3	26	1.0
Age at Delivery (years) ^a,c^									
	<18 years old	246	3.7	43	3.7	30	4.3	88	3.2
	18-19 years old	465	6.9	94	8.0	53	7.7	190	7.0
	20-24 years old	1,517	22.7	256	21.9	175	25.3	694	25.5
	25-29 years old	1,781	26.6	381	32.6	172	24.8	728	26.7
	30-34 years old	1,741	26.0	244	20.9	179	25.8	615	22.6
	35-39 years old	810	12.1	111	9.5	73	10.5	342	12.5
	40-44 years old	126	1.9	38	3.3	11	1.6	64	2.4
	45-49 years old	11	0.2	3	0.3	0	0.0	6	0.2
	>49 Years old	1	0.0	0	0.0	0	0.0	0	0.0
Study Center^a,b,c^									
	Arkansas	836	12.5	145	12.4	66	9.5	306	11.2
	California	839	12.5	232	19.8	114	16.5	437	16.0
	Georgia	749	11.2	138	11.8	73	10.5	302	11.1
	Iowa	849	12.7	73	6.2	89	12.8	382	14.0
	Massachusetts	568	8.5	68	5.8	78	11.3	189	6.9
	North Carolina	596	8.9	70	6.0	49	7.1	246	9.0
	New Jersey	772	11.5	162	13.9	92	13.3	343	12.6
	New York	720	10.8	138	11.8	73	10.5	328	12.0
	Texas	405	6.1	70	6.0	13	1.9	99	3.6
	Utah	364	5.4	74	6.3	46	6.6	95	3.5
Maternal Body Mass Index^a,c^ (kg/m^2^)									
	Underweight (BMI <18)	353	5.3	48	4.1	38	5.5	183	6.7
	Normal weight (18.5 -25)	3,585	53.5	544	46.5	337	48.6	1,376	50.5
	Overweight (25–30)	1,443	21.5	243	20.8	164	23.7	569	20.9
	Obese (≥30)	1,037	15.5	259	22.1	121	17.5	479	17.6
	Missing	280	4.2	76	6.5	33	4.8	120	4.4
Folic Acid Supplement Use^c^									
	No	3,053	45.6	552	47.2	305	44.0	1,330	48.8
	Yes	3,480	52.0	577	49.3	366	52.8	1,340	49.1
	Missing	165	2.5	41	3.5	22	3.2	57	2.1

^a^ Statistically significant difference in covariate distribution between control mothers and mothers of offspring with a NTD.

^b^ Statistically significant difference in covariate distribution between control mothers and mothers of offspring with a limb malformation.

^c^ Statistically significant difference in covariate distribution between control mothers and mothers of offspring with an orofacial cleft.

### Outcomes

This study focused on three major classes of birth defects: neural tube defects, orofacial clefts and limb malformations. Specific neural tube defects included anencephaly, spina bifida and encephalocele. Orofacial clefts included cleft lip without cleft palate, cleft lip with cleft palate, cleft palate, and cleft lip with or without cleft palate. Limb malformations included longitudinal limb deficiency, longitudinal preaxial limb deficiency (a subcategory of longitudinal limb deficiency), transverse limb deficiency, and intercalary limb deficiency. Cases were identified through one of ten birth defect registries. Most of the registry sites include prenatal diagnosis and terminations (including Arkansas, California, Georgia, Iowa, North Carolina, Texas, and Utah) to avoid the potential for selection bias due to possible differences between mothers who choose to terminate their pregnancies and those who do not. At each site, cases were checked for validity by a clinician who reviewed the abstracted records using a standardized protocol [18]. Cases from all sites were further classified by a clinical geneticist before study analyses began. The institutional review boards (for the protection of human subjects) at each site and the Centers for Disease Control and Prevention approved the NBDPS study protocol, and the institutional review boards of Texas A&M University and the Texas Department of State Health Services also approved this project on maternal dietary intake of nitrates, nitrites, and nitrosamines and birth defects.

### Dietary intake of nitrate, nitrite, and nitrosamines

The exposures of interest included dietary nitrate (mg/day), nitrite (mg/day) and nitrosamines (μg/day). Daily consumption of nitrates, nitrites and nitrosamines was estimated using a 58-item food frequency questionnaire based on the Willett Food Frequency Questionnaire [19,20]. The NDBPS questionnaire included additional region-specific food items such as avocados, raw chili peppers, salsa, tortillas, cantaloupe, and refried beans that are commonly consumed in this population.

The nitrate, nitrite and nitrosamine content of each food item was estimated based on an extensive literature review reported by Griesenbeck et al. [21]. Total daily consumption of each compound was calculated by multiplying the number of servings of each food item eaten each day by the estimated content of nitrate, nitrite, and nitrosamines in the standard serving size and summing over all food items. Due to the endogenous conversion of nitrate to nitrite in the saliva and stomach, 5% of total dietary nitrate consumption was added to estimate total dietary nitrite consumption. Quartiles of nitrate, nitrite from animal and plant sources separately, total nitrite, and nitrosamine intake were based on the distributions of control mothers’ daily consumption of these compounds, with the lowest quartile of each compound used as the referent category in all analyses.

### Statistical methods

Data from dietary recall questionnaires often includes some degree of measurement error. Since this study did not include a “gold standard” for comparison purposes, the effects of measurement error were evaluated using the Simulation Extrapolation (SIMEX) algorithm [22] and hypothetically varying the amount of measurement error included in the model from no error to a multiplicative factor of 1.6 (60% additional variability) in increments of 0.10. No substantive differences were identified in terms of statistical significance or magnitude of effect size; therefore, all subsequent statistical models were conducted using logistic regression with maximum likelihood estimation as implemented in Stata 11 [23] and SAS 9.2 [24] to estimate the odds ratios and respective 95% confidence limits for the specific defects. Each specific type of birth defect was compared with controls only, and pregnancies with other kinds of defects were not included as controls.

### Covariates and model selection

Several demographic, dietary, and behavioral characteristics including race/ethnicity, state of residence, dietary folate (μg/day), maternal education (years in school), dietary fat (percent of calories from fat), maternal household income, pre-pregnancy body mass index (BMI) (kg/m^2^), use of folic acid-containing supplements, multivitamin use, and age at conception were found in a previous study to be associated with dietary intake of nitrates, nitrites and nitrosamines in the control group mothers [25]. These variables were examined for potential confounding effects using a backward selection procedure where covariates were retained if their removal changed any of the three exposure quartile parameters by more than 10%. In the interest of interpretability, covariates were retained within the broad classes of birth defects (i.e. NTD, orofacial defect and limb malformation) if evidence of potential confounding was identified in any of the constituent birth defect sub-categories.

## Results

Table 1 displays the demographic characteristics of the control group as well as each of the broad classes of birth defects. A majority of the participants were of non-Hispanic white race/ethnicity with the second most common group being of Hispanic origin. The mothers were relatively well educated with the majority having a high school and/or college degree. Roughly half of all mothers delivered their children between the ages of 20 and 29 and nearly three quarters delivered between the ages of 20 and 34. Approximately half of all case and control mothers had a body mass index (BMI) in the normal range while nearly one third had a BMI in the overweight and obese range. Roughly half of all case and control mothers reported using folic acid supplements during the first month of pregnancy.

Statistical significance in Table 1 is based on the pairwise comparison of each birth defect class case mothers with the control mothers. The distributions of race/ethnicity, maternal education, age at delivery, study center, and maternal BMI for mothers of offspring with NTDs were significantly different than the distributions of the control mothers. The distributions of race/ethnicity and study center for mothers of offspring with limb malformations were significantly different than the distributions of the control mothers. The distributions of race/ethnicity, maternal education, age at delivery, study center, maternal BMI and folic acid use for mothers of offspring with oral clefts were significantly different than the distributions of the control mothers.

The descriptive statistics for daily consumption of nitrates, total nitrites, animal nitrites, plant nitrites and nitrosamines are shown in Table 2 for mothers of children with NTDs. The results of the unadjusted and adjusted logistic regression models for NTDs are shown in Table 3. Compared to the first quartile of nitrate consumption, a significant relationship was identified for the fourth quartile for spina bifida (uOR = 0.77, 95% confidence interval = 0.61-0.97), but this association was not statistically significant after adjustment for covariates.

**Table 2:** Descriptive statistics of dietary nitrate, nitrite, and nitrosamine intake and neural tube defects in offspring

				**N (%)**			
	** Outcome**	**N**	**Mean (SD)**	**Quartile 1**	**Quartile 2**	**Quartile 3**	**Quartile 4**
Nitrate (mg/day)							
	Anencephaly	319	48.02 (32.99)	90 (28.2)	84 (26.3)	65 (20.4)	80 (25.1)
	Spina bifida	658	49.83 (43.40)	179 (27.2)	168 (25.5)	172 (26.1)	139 (21.1)
	Encephalocele	127	49.75 (36.88)	35 (27.6)	28 (22.0)	37 (29.1)	27 (21.3)
Total Nitrite (mg/day)							
	Anencephaly	319	1.88 (0.99)	74 (23.2)	63 (19.7)	86 (27.0)	96 (30.1)
	Spina bifida	662	1.85 (1.05)	157 (23.7)	153 (23.1)	165 (24.9)	187 (28.2)
	Encephalocele	127	1.84 (1.04)	32 (25.2)	31 (24.4)	28 (22.0)	36 (28.3)
Animal Nitrite (mg/day)							
	Anencephaly	320	1.15 (0.70)	71 (22.2)	71 (22.2)	89 (27.8)	89 (27.8)
	Spina bifida	673	1.14 (0.79)	157 (23.3)	166 (24.7)	180 (26.7)	170 (25.3)
	Encephalocele	128	1.20 (0.89)	37 (28.9)	23 (18.0)	31 (24.2)	37 (28.9)
Plant Nitrite (mg/day)							
	Anencephaly	319	0.74 (0.51)	76 (23.8)	84 (26.3)	65 (20.4)	94 (29.5)
	Spina bifida	666	0.72 (0.49)	170 (25.5)	162 (24.3)	138 (20.7)	196 (29.4)
	Encephalocele	129	0.66 (0.37)	29 (22.5)	31 (24.0)	38 (29.5)	31 (24.0)
Nitrosamine (μg/day)							
	Anencephaly	315	0.53 (0.30)	77 (24.4)	77 (24.4)	84 (26.7)	77 (24.4)
	Spina bifida	664	0.55 (0.34)	181 (27.3)	150 (22.6)	157 (23.6)	176 (26.5)
	Encephalocele	127	0.52 (0.32)	34 (26.8)	34 (26.8)	26 (20.5)	33 (26.0)

**Table 3:** Maternal dietary intake of nitrate, nitrite, and nitrosamines and neural tube defects in offspring

		**Unadjusted odds ratios (95% CI)**			Adjusted odds ratios* (95% CI)		
	** Outcome**	**Quartile 1 vs Quartile 2**	**Quartile 1 vs Quartile 3**	**Quartile 1 vs Quartile 4**	**Quartile 1 vs Quartile 2**	**Quartile 1 vs Quartile 3**	**Quartile 1 vs Quartile 4**
Nitrate							
	anencephaly	0.93 (0.68-1.27)	0.72 (0.52-1.01)	0.90 (0.66-1.23)	0.97 (0.71-1.34)	0.81 (0.56-1.17)	0.99 (0.67-1.45)
	spina bifida	0.93 (0.74-1.16)	0.96 (0.77-1.20)	**0.77 (0.61-0.97)**	0.93 (0.73-1.17)	0.98 (0.77-1.26)	0.78 (0.59-1.04)
	encephalocele	0.80 (0.48-1.32)	1.00 (0.62-1.60)	0.74 (0.45-1.24)	0.75 (0.45-1.26)	0.87 (0.52-1.48)	0.59 (0.32-1.08)
Total Nitrite							
	anencephaly	0.83 (0.59-1.18)	1.11 (0.80-1.54)	1.31 (0.96-1.79)	0.82 (0.57-1.18)	1.04 (0.73-1.49)	1.06 (0.69-1.64)
	spina bifida	0.96 (0.76-1.22)	1.08 (0.86-1.36)	1.18 (0.94-1.48)	0.96 (0.76-1.23)	1.02 (0.79-1.32)	1.00 (0.73-1.38)
	encephalocele	1.00 (0.60-1.66)	0.95 (0.56-1.59)	1.20 (0.74-1.97)	0.97 (0.57-1.64)	0.83 (0.47-1.47)	0.96 (0.49-1.89)
Animal Nitrite							
	anencephaly	1.01 (0.72-1.43)	1.27 (0.92-1.76)	1.27 (0.91-1.75)	1.00 (0.71-1.42)	1.22 (0.86-1.73)	1.12 (0.76-1.67)
	spina bifida	1.07 (0.85-1.34)	1.16 (0.92-1.46)	1.07 (0.85-1.35)	1.06 (0.83-1.34)	1.12 (0.88-1.43)	1.00 (0.75-1.33)
	encephalocele	0.59 (0.34-1.02)	0.88 (0.54-1.43)	1.04 (0.66-1.66)	0.59 (0.34-1.03)	0.86 (0.51-1.44)	0.98 (0.55-1.74)
Plant Nitrite							
	anencephaly	1.13 (0.81-1.56)	0.87 (0.62-1.23)	1.26 (0.92-1.73)	1.18 (0.84-1.66)	0.86 (0.59-1.26)	0.95 (0.60-1.50)
	spina bifida	0.95 (0.75-1.19)	0.82 (0.65-1.05)	1.14 (0.92-1.43)	0.94 (0.74-1.20)	0.81 (0.62-1.05)	0.90 (0.65-1.24)
	encephalocele	1.02 (0.61-1.71)	1.23 (0.75-2.02)	1.05 (0.63-1.76)	0.99 (0.58-1.70)	1.04 (0.59-1.82)	0.61 (0.30-1.25)
Nitrosamine							
	anencephaly	1.01 (0.73-1.40)	1.10 (0.80-1.52)	1.03 (0.74-1.43)	1.10 (0.78-1.55)	1.18 (0.82-1.68)	1.06 (0.70-1.60)
	spina bifida	0.81 (0.65-1.03)	0.87 (0.69-1.09)	0.98 (0.78-1.22)	0.82 (0.64-1.04)	0.85 (0.66-1.10)	0.91 (0.69-1.21)
	encephalocele	0.98 (0.60-1.60)	0.79 (0.47-1.33)	1.03 (0.63-1.68)	1.01 (0.60-1.68)	0.82 (0.47-1.46)	1.13 (0.60-2.11)

* Logistic regression models adjusted for energy intake, maternal race/ethnicity, dietary folate intake, folic acid supplementation, and dietary fat intake.

The descriptive statistics for diet of mothers of children with orofacial defects are reported in Table 4 and the results of the logistic regression models are reported in Table 5. In the unadjusted logistic regression models, statistically significant relationships were identified between the second quartile of nitrite intake and cleft palate (uOR = 0.82, 0.67-0.99), between the second quartile of nitrosamine intake and cleft lip without cleft palate (uOR = 0.74, 0.58-0.94), between the third quartile of nitrosamine intake and cleft lip without cleft palate (uOR = 0.72, 0.56-0.92), between the second quartile of nitrosamine intake and cleft lip with or without cleft palate (uOR = 0.83, 0.72-0.97), between the third quartile of nitrosamine intake and cleft lip with or without cleft palate (uOR = 0.82, 0.71-0.96) and between the fourth quartile of nitrosamine intake and cleft palate (uOR = 0.77, 0.64-0.94). However, none of these relationships was statistically significant after adjustment for covariates. For the adjusted logistic regression models, a significant relationship was identified between the second quartile of nitrosamine intake and cleft palate (aOR = 0.78, 0.64-0.95).

**Table 4:** Descriptive statistics of dietary nitrate, nitrite, and nitrosamine intake and oral cleft defects in offspring

				**N (%)**			
	** Outcome**	**N**	**Mean (SD)**	**Quartile 1**	**Quartile 2**	**Quartile 3**	**Quartile 4**
Nitrate (mg/day)							
	Cleft lip only	613	50.31 (52.16)	165 (26.9)	162 (26.4)	139 (22.7)	147 (24.0)
	Cleft lip with cleft palate	1133	48.65 (37.08)	293 (25.9)	309 (27.3)	273 (24.1)	258 (22.8)
	Cleft palate only	910	49.86 (36.66)	230 (25.3)	233 (25.6)	224 (24.6)	223 (24.5)
	Cleft lip with or without cleft palate	1746	49.23 (42.97)	458 (26.2)	471 (27.0)	412 (23.6)	405 (23.2)
Nitrite (mg/day)							
	Cleft lip only	614	1.75 (0.97)	166 (27.0)	136 (22.1)	158 (25.7)	154 (25.1)
	Cleft lip with cleft palate	1133	1.88 (1.06)	263 (23.2)	262 (23.1)	275 (24.3)	333 (29.4)
	Cleft palate only	910	1.72 (0.96)	257 (28.2)	211 (23.2)	228 (25.1)	214 (23.5)
	Cleft lip with or without cleft palate	1747	1.83 (1.03)	429 (24.6)	398 (22.8)	433 (24.8)	487 (27.9)
Animal Nitrite (mg/day)							
	Cleft lip only	615	1.11 (0.75)	151 (24.6)	149 (24.2)	144 (23.4)	171 (27.8)
	Cleft lip with cleft palate	1140	1.17 (0.78)	262 (23.0)	275 (24.1)	269 (23.6)	334 (29.3)
	Cleft palate only	918	1.07 (0.71)	249 (27.1)	219 (23.9)	230 (25.1)	220 (24.0)
	Cleft lip with or without cleft palate	1755	1.15 (0.77)	413 (23.5)	424 (24.2)	413 (23.5)	505 (28.8)
Plant Nitrite (mg/day)							
	Cleft lip only	614	0.64 (0.38)	153 (24.9)	160 (26.1)	162 (26.4)	139 (22.6)
	Cleft lip with cleft palate	1140	0.71 (0.49)	280 (24.6)	274 (24.0)	261 (22.9)	325 (28.5)
	Cleft palate only	914	0.65 (0.41)	242 (26.5)	221 (24.2)	251 (27.5)	200 (21.9)
	Cleft lip with or without cleft palate	1754	0.69 (0.45)	433 (24.7)	434 (24.7)	423 (24.1)	464 (26.5)
Nitrosamine (μg/day)							
	Cleft lip only	614	0.72 (4.60)	169 (27.5)	126 (20.5)	171 (27.9)	148 (24.1)
	Cleft lip with cleft palate	1133	0.53 (0.31)	302 (26.7)	269 (23.7)	283 (25.0)	279 (24.6)
	Cleft palate only	908	0.52 (0.30)	264 (29.1)	226 (24.9)	209 (23.0)	209 (23.0)
	Cleft lip with or without cleft palate	1747	0.60 (2.74)	471 (27.0)	395 (22.6)	454 (26.0)	427 (24.4)

**Table 5:** Maternal dietary intake of nitrate, nitrite, and nitrosamines and oral cleft defects in offspring

		**Unadjusted odds ratios (95% CI)**			Adjusted odds ratios* (95% CI)		
	**Outcome**	**Quartile 1 vs Quartile 2**	**Quartile 1 vs Quartile 3**	**Quartile 1 vs Quartile 4**	**Quartile 1 vs Quartile 2**	**Quartile 1 vs Quartile 3**	**Quartile 1 vs Quartile 4**
Nitrate							
	Cleft lip only	1.00 (0.80-1.26)	0.85 (0.67-1.08)	0.90 (0.71-1.14)	1.01 (0.80-1.28)	0.89 (0.68-1.15)	1.01 (0.76-1.33)
	Cleft lip with cleft plate	1.05 (0.88-1.25)	0.93 (0.77-1.11)	0.89 (0.74-1.07)	1.07 (0.89-1.28)	0.96 (0.79-1.16)	0.90 (0.73-1.13)
	Cleft palate only	1.02 (0.83-1.24)	0.99 (0.81-1.20)	0.98 (0.81-1.20)	1.06 (0.86-1.29)	1.05 (0.85-1.30)	1.15 (0.91-1.46)
	Cleft lip with or without cleft palate	1.04 (0.89-1.20)	0.90 (0.77-1.05)	0.89 (0.77-1.04)	1.04 (0.90-1.22)	0.93 (0.79-1.09)	0.93 (0.78-1.12)
Nitrite							
	Cleft lip only	0.82 (0.64-1.04)	0.94 (0.75-1.19)	0.92 (0.73-1.17)	0.85 (0.67-1.08)	1.03 (0.80-1.33)	1.11 (0.81-1.51)
	Cleft lip with cleft plate	0.99 (0.82-1.19)	1.04 (0.86-1.25)	1.25 (1.04-1.49)	1.02 (0.85-1.24)	1.02 (0.84-1.25)	1.12 (0.88-1.42)
	Cleft palate only	0.82 (0.67-0.99)	0.87 (0.72-1.06)	0.83 (0.69-1.01)	0.85 (0.70-1.04)	0.97 (0.79-1.20)	1.02 (0.78-1.32)
	Cleft lip with or without cleft palate	0.92 (0.79-1.08)	1.00 (0.86-1.16)	1.12 (0.97-1.30)	0.95 (0.81-1.11)	1.02 (0.86-1.20)	1.10 (0.91-1.35)
Animal Nitrite							
	Cleft lip only	0.98 (0.77-1.24)	0.92 (0.72-1.17)	1.12 (0.89-1.42)	1.00 (0.78-1.27)	1.00 (0.78-1.29)	1.32 (1.01-1.72)
	Cleft lip with cleft plate	1.03 (0.86-1.24)	1.01 (0.84-1.21)	1.24 (1.04-1.48)	1.06 (0.88-1.28)	1.03 (0.85-1.24)	1.22 (0.99-1.49)
	Cleft palate only	0.86 (0.71-1.05)	0.90 (0.74-1.10)	0.87 (0.72-1.06)	0.89 (0.73-1.08)	0.97 (0.79-1.19)	1.01 (0.80-1.27)
	Cleft lip with or without cleft palate	1.01 (0.87-1.18)	0.97 (0.84-1.14)	1.20 (1.04-1.39)	1.03 (0.88-1.21)	1.01 (0.86-1.19)	1.24 (1.05-1.48)
Plant Nitrite							
	Cleft lip only	1.02 (0.81-1.29)	1.05 (0.83-1.32)	0.91 (0.71-1.15)	1.09 (0.85-1.39)	1.15 (0.89-1.50)	1.04 (0.74-1.46)
	Cleft lip with cleft plate	0.98 (0.82-1.17)	0.92 (0.77-1.11)	1.16 (0.98-1.39)	1.03 (0.85-1.24)	0.95 (0.77-1.16)	0.98 (0.76-1.27)
	Cleft palate only	0.92 (0.75-1.12)	1.03 (0.85-1.24)	0.84 (0.69-1.03)	0.97 (0.79-1.19)	1.15 (0.93-1.43)	1.01 (0.76-1.33)
	Cleft lip with or without cleft palate	1.00 (0.86-1.16)	0.97 (0.83-1.12)	1.07 (0.92-1.24)	1.05 (0.89-1.23)	1.02 (0.86-1.20)	1.00 (0.81-1.24)
Nitrosamine							
	Cleft lip only	0.74 (0.58-0.94)	0.72 (0.56-0.92)	1.02 (0.81-1.27)	0.87 (0.69-1.09)	0.99 (0.78-1.26)	0.85 (0.64-1.13)
	Cleft lip with cleft plate	0.89 (0.74-1.06)	0.89 (0.74-1.06)	0.93 (0.78-1.11)	0.90 (0.75-1.08)	0.95 (0.78-1.15)	0.89 (0.71-1.10)
	Cleft palate only	0.85 (0.70-1.03)	0.86 (0.71-1.05)	0.77 (0.64-0.94)	0.78 (0.64-0.95)	0.85 (0.69-1.05)	0.88 (0.69-1.11)
	Cleft lip with or without cleft palate	0.83 (0.72-0.97)	0.83 (0.71-0.96)	0.96 (0.83-1.11)	0.89 (0.76-1.03)	0.96 (0.82-1.13)	0.87 (0.73-1.04)

*Logistic regression models adjusted for energy intake, maternal race/ethnicity, maternal education, dietary folate intake, and study center.

The descriptive statistics for diet for mothers of children with limb malformations are reported in Table 6 and the results of the logistic regression models are reported in Table 7. In the unadjusted logistic regression models with the lowest quartile of intake serving as the referent category, a significant relationship was noted for the second quartile of total nitrite consumption and longitudinal limb deficiency (uOR = 0.67, 0.46-0.98), but this result was not significant after adjustment for covariates. In the adjusted logistic regression models, there was a significant relationship between the fourth quartile of total nitrite consumption and intercalary limb deficiency (aOR = 4.70, 1.23-17.93).

**Table 6:** Descriptive statistics of dietary nitrate, nitrite, and nitrosamine intake and limb deficiency defects in offspring

				**N (%)**			
	**Limb deficiency defect**	**N**	**Mean (SD)**	**Quartile 1**	**Quartile 2**	**Quartile 3**	**Quartile 4**
Nitrate (mg/day)							
	Longitudinal	245	47.12 (31.52)	73 (29.8)	55 (22.4)	60 (24.5)	57 (23.3)
	Transverse	379	46.97 (33.93)	101 (26.6)	105 (27.7)	95 (25.1)	78 (20.6)
	Intercalary	34	45.84 (24.04)	10 (29.4)	7 (20.6)	7 (20.6)	10 (29.4)
	Preaxial*	143	45.19 (30.13)	46 (32.2)	32 (22.4)	34 (23.8)	31 (21.7)
Nitrite (mg/day)							
	Longitudinal	245	1.76 (0.96)	73 (29.8)	50 (20.4)	54 (22.0)	68 (27.8)
	Transverse	379	1.80 (1.00)	95 (25.1)	102 (26.9)	86 (22.7)	96 (25.3)
	Intercalary	34	1.86 (0.88)	5 (14.7)	9 (26.5)	7 (20.6)	13 (38.2)
	Preaxial*	143	1.78 (1.02)	42 (29.4)	33 (23.1)	31 (21.7)	37 (25.9)
Animal Nitrite (mg/day)							
	Longitudinal	247	1.12 (0.79)	66 (26.7)	59 (23.9)	64 (25.9)	58 (23.5)
	Transverse	381	1.12 (0.77)	99 (26.0)	94 (24.7)	90 (23.6)	98 (25.7)
	Intercalary	34	1.26 (0.82)	5 (14.7)	7 (20.6)	9 (26.5)	13 (38.2)
	Preaxial*	145	1.15 (0.87)	38 (26.2)	37 (25.5)	36 (24.8)	34 (23.4)
Plant Nitrite (mg/day)							
	Longitudinal	247	0.64 (0.36)	64 (25.9)	63 (25.5)	56 (22.7)	64 (25.9)
	Transverse	381	0.68 (0.42)	101 (26.5)	89 (23.4)	83 (21.8)	108 (28.3)
	Intercalary	34	0.60 (0.29)	8 (23.5)	9 (26.5)	10 (29.4)	7 (20.6)
	Preaxial*	145	0.63 (0.34)	37 (25.5)	39 (26.9)	33 (22.8)	36 (24.8)
Nitrosamine (μg/day)							
	Longitudinal	245	0.56 (0.46)	65 (26.5)	62 (25.3)	55 (22.4)	63 (25.7)
	Transverse	380	0.82 (5.82)	100 (26.3)	99 (26.1)	91 (23.9)	90 (23.7)
	Intercalary	34	0.63 (0.30)	4 (11.8)	9 (26.5)	11 (32.4)	10 (29.4)
	Preaxial*	143	0.52 (0.31)	41 (28.7)	34 (23.8)	33 (23.1)	35 (24.5)

* Subcategory of longitudinal limb deficiency.

**Table 7:** Maternal dietary intake of nitrate, nitrite, and nitrosamines and limb deficiency defects in offspring

		**Unadjusted odds ratios (95% CI)**			Adjusted odds ratios** (95% CI)		
	**Limb deficiency defect**	**Quartile 1 vs Quartile 2**	**Quartile 1 vs Quartile 3**	**Quartile 1 vs Quartile 4**	**Quartile 1 vs Quartile 2**	**Quartile 1 vs Quartile 3**	**Quartile 1 vs Quartile 4**
Nitrate							
	Longitudinal	0.75 (0.52-1.07)	0.79 (0.55-1.13)	0.78 (0.55-1.11)	0.74 (0.51-1.06)	0.82 (0.56-1.19)	0.79 (0.52-1.20)
	Transverse	1.07 (0.80-1.43)	0.97 (0.72-1.30)	0.80 (0.59-1.09)	1.02 (0.76-1.36)	0.91 (0.66-1.24)	0.73 (0.51-1.04)
	Intercalary	0.70 (0.27-1.84)	0.70 (0.27-1.84)	1.00 (0.42-2.41)	0.76 (0.28-2.06)	0.90 (0.31-2.60)	1.40 (0.48-4.10)
	Preaxial*	0.69 (0.43-1.10)	0.71 (0.45-1.12)	0.69 (0.43-1.10)	0.67 (0.42-1.07)	0.72 (0.44-1.18)	0.65 (0.37-1.13)
Nitrite							
	Longitudinal	0.67 (0.46-0.98)	0.77 (0.53-1.10)	0.93 (0.66-1.31)	0.69 (0.48-1.01)	0.75 (0.51-1.12)	0.95 (0.59-1.52)
	Transverse	1.08 (0.81-1.45)	0.94 (0.70-1.28)	1.04 (0.77-1.39)	1.10 (0.82-1.48)	0.91 (0.65-1.27)	0.95 (0.63-1.42)
	Intercalary	1.79 (0.60-5.36)	1.41 (0.45-4.46)	2.61 (0.93-7.33)	2.29 (0.74-7.07)	1.92 (0.56-6.59)	4.70 (1.23-17.93)
	Preaxial*	0.80 (0.50-1.28)	0.78 (0.49-1.26)	0.90 (0.57-1.42)	0.74 (0.46-1.20)	0.65 (0.39-1.09)	0.64 (0.34-1.20)
Animal Nitrite							
	Longitudinal	0.87 (0.60-1.25)	0.99 (0.69-1.41)	0.89 (0.62-1.28)	0.91 (0.63-1.31)	0.95 (0.65-1.38)	0.83 (0.53-1.29)
	Transverse	0.91 (0.67-1.22)	0.93 (0.69-1.25)	0.99 (0.74-1.33)	1.01 (0.75-1.37)	0.98 (0.72-1.34)	1.13 (0.79-1.61)
	Intercalary	1.41 (0.45-4.47)	1.84 (0.61-5.49)	2.63 (0.94-7.41)	1.56 (0.48-5.01)	1.94 (0.61-6.15)	2.73 (0.80-9.37)
	Preaxial*	0.96 (0.60-1.53)	0.97 (0.60-1.54)	0.93 (0.58-1.49)	0.96 (0.60-1.53)	0.84 (0.51-1.38)	0.69 (0.39-1.23)
Plant Nitrite							
	Longitudinal	0.96 (0.67-1.37)	0.87 (0.60-1.26)	0.97 (0.68-1.39)	1.00 (0.69-1.44)	0.91 (0.60-1.38)	1.06 (0.64-1.74)
	Transverse	0.87 (0.64-1.17)	0.81 (0.60-1.10)	1.08 (0.81-1.43)	0.88 (0.65-1.20)	0.82 (0.58-1.14)	0.92 (0.61-1.38)
	Intercalary	1.11 (0.43-2.89)	1.25 (0.49-3.16)	0.87 (0.31-2.39)	1.36 (0.50-3.70)	1.81 (0.62-5.28)	1.61 (0.40-6.47)
	Preaxial*	1.05 (0.66-1.66)	0.89 (0.55-1.43)	0.96 (0.60-1.54)	1.09 (0.67-1.75)	0.91 (0.53-1.55)	0.90 (0.47-1.73)
Nitrosamine							
	Longitudinal	0.95 (0.67-1.37)	0.88 (0.61-1.27)	1.01 (0.71-1.45)	0.99 (0.68-1.42)	0.86 (0.58-1.29)	1.03 (0.65-1.61)
	Transverse	1.01 (0.76-1.36)	0.95 (0.71-1.28)	0.92 (0.68-1.24)	1.02 (0.76-1.38)	0.96 (0.70-1.33)	1.00 (0.69-1.46)
	Intercalary	2.26 (0.69-7.34)	2.76 (0.88-8.70)	2.57 (0.80-8.20)	2.50 (0.74-8.40)	3.05 (0.88-10.56)	2.75 (0.68-11.08)
	Preaxial*	0.80 (0.50-1.28)	0.83 (0.52-1.32)	0.90 (0.57-1.42)	0.83 (0.52-1.34)	0.76 (0.45-1.28)	0.77 (0.43-1.40)

* Subcategory of longitudinal limb deficiency.

** Logistic regression models adjusted for energy intake, maternal race/ethnicity, maternal education, dietary folate intake, dietary fat intake, and study center.

## Discussion

This study explored the relationship between maternal consumption of dietary nitrates, total nitrites, nitrites (from both animal and plant sources) and nitrosamines and specific NTDs, orofacial clefts and limb malformations in their offspring. The primary strength of the study is its large and very well-characterized sample. It is the largest study to date to investigate the relation between estimated maternal intake of dietary nitrate, nitrite, and nitrosamines and neural tube defects and examines several other types of birth defects in relation to these exposures that have not been examined before. Overall, estimated dietary intake of these compounds did not appear to be significant risk factors for neural tube, oral cleft, or limb deficiency defects.

Croen et al. [1] also found no compelling associations between maternal dietary intake of nitrates, nitrites, and nitrosamines and neural tube defects in a California study population, with most odds ratios for neural tube defects slightly below 1.00 in the second, third, and fourth quartiles compared with the first quartile of intake. Using tertiles instead of quartiles of intake, Brender et al. [2] noted odds ratios of 0.8 and 0.9 for the upper two tertiles of dietary nitrite and for total nitrite, 0.9 and 0.8. Neither study reported findings of the relation between dietary intake of these compounds and neural tube defects by specific phenotype.

Of interest to this study, three studies found dietary nitrite and total nitrite to modify the association between nitrosatable drug use and birth defects in offspring. In a study of Mexican American women who resided in Texas counties bordering Mexico, nitrosatable drug use was associated with these defects in the upper two tertiles of nitrite and total nitrite intake, but not in the lowest tertile of intake [2]. These findings were corroborated in the National Birth Defects Prevention Study population [3] in which the strongest associations between secondary/tertiary amine drug exposure and anencephaly and spina bifida were noted in the upper two tertiles of dietary nitrite and total nitrite intake. Associations for cleft palate and several types of heart defects were also stronger in offspring of NBDPS participants who had the highest estimated total nitrite intake [15]. These findings are consistent with results of an experimental study with mice exposed to ethylenethiourea (a nitrosatable compound) and nitrite [26]. Malformations were observed when these compounds were administered together, but not separately. With the dose of the nitrosatable compound held constant, the percentage of malformations increased as the dose of nitrite increased, indicating that the combined effects of these compounds might be due to the nitrosation products formed within the stomach.

One possible limitation of our study is the potential for measurement error in the self-reported food frequency questionnaire. Because data from food frequency questionnaires are known to be measured with error [19,20], logistic regression models were estimated using the SIMEX algorithm. Since there was no “gold standard” available to quantify the amount of measurement error in the data, extra error was considered by adding 0% to 60% additional variance in increments of 10%. There was no evidence that even the highest levels of measurement error made any substantive different in the results so maximum likelihood estimation was used for all subsequent modeling. It is possible that the inability to explicitly quantify the degree of measurement error in the exposure variables could bias the results towards the null. However, any bias due to measurement error should be non-differential because the mothers were not aware of the nitrate, nitrite and nitrosamine content in the foods they consumed when they completed the dietary recall questionnaire.

In this study, we focused on dietary contributions to daily intake of nitrate, nitrite, and nitrosamines, although drinking water is another potential source of nitrate intake. On the other hand, the World Health Organization noted, in a recent review, that the contribution of drinking water to nitrate intake is usually less than 14% [27].

## Conclusion

Over 165 unadjusted and 165 adjusted logistic regression models were fit in the course of this analysis, and only four adjusted odds ratios had confidence intervals that did not include the null value. Though no explicit adjustment for multiple comparisons was made, it is likely that the four significant results would not remain so if adjusted. Given the small number of significant results relative to the number of models considered and the modest effects for the non-significant results, it seems reasonable to conclude that there is insufficient evidence to suggest any relationship between dietary intake of nitrates, nitrosamines, total nitrites or nitrates from animal or plant sources and any of the three groups of birth defects including NTDs, orofacial clefts and limb malformations. Because nitrite can react with nitrosatable compounds within the stomach to form N-nitroso compounds, further studies are recommended on the relation between the interaction of dietary nitrites with nitrosatable drugs and adverse pregnancy outcomes.

## Abbreviations

uOR: Unadjusted odds ratio; aOR: Adjusted odds ratio; B1P1: One month prior through one month post-conception; CI: Confidence interval; EDD: Estimated delivery date; NBDPS: National Birth Defects Prevention Study; NTD: Neural tube defect; OR: Odds ratio.

## Competing interests

The authors declare that they have no competing interest.

## Authors’ contributions

JCH analyzed the data and prepared the manuscript, JDB conceived of the study and serves as principal investigator of the project, assisted in the data analysis and preparation of the manuscript, QZ analyzed the data and assisted in preparing the manuscript. JRS provided guidance on the nutritional aspects of the project and assisted with the manuscript, AV and MS assisted with data analysis and manuscript development. JSG developed the estimates of nitrates, nitrites, and nitrosamines from the food frequency and provided the initial analyses on maternal characteristics related to estimated higher dietary consumption of these compounds. LS, PHL, MAC, PAR, and PJW provided input into the study design and final paper. All authors approved the final draft.

## Authors’ information

Supported by the National Institutes of Health, National institute for Environmental Health Sciences (5RO1ES015634 and 3R01ES015634-03S1).
